# Perfluorooctane sulfonate (PFOS) and perfluorooctanoic acid (PFOA) induce epigenetic alterations and promote human breast cell carcinogenesis in vitro

**DOI:** 10.1007/s00204-020-02848-6

**Published:** 2020-07-22

**Authors:** Paula Pierozan, Daiane Cattani, Oskar Karlsson

**Affiliations:** Science for Life Laboratory, Department of Environmental Sciences, Stockholm University, 114 18 Stockholm, Sweden

**Keywords:** PFOS, PFOA, Cell transformation, Cyclin D1, p21, Histone modification, DNA methylation

## Abstract

Gene–environment interactions are involved in the development of breast cancer, the tumor type that accounts for the majority of the cancer-related deaths among women. Here, we demonstrate that exposure to PFOS (10 µM) and PFOA (100 µM)—two contaminants ubiquitously found in human blood—for 72 h induced breast epithelial cell (MCF-10A cell line) proliferation and alteration of regulatory cell-cycle proteins (cyclin D1, CDK6, p21, p53, p27, ERK 1/2 and p38) that persisted after a multitude of cell divisions. The contaminants also promoted cell migration and invasion by reducing the levels of E-cadherin, occludin and β-integrin in the unexposed daughter cells. The compounds further induced an increase in global DNA methylation and differentially altered histone modifications, epigenetic mechanisms implicated in tumorigenesis. This mechanistic evidence for PFOS- and PFOA-induced malignant transformation of human breast cells supports a role of these abundant contaminants in the development and progression of breast cancer. Increased knowledge of contaminant-induced effects and their contribution to breast tumorigenesis is important for a better understanding of gene–environment interactions in the etiology of breast cancer.

## Introduction

Breast cancer is the most commonly diagnosed cancer among women in the 35–54 age range worldwide and its incidence has increased in almost all Western countries (Brody and Rudel 2003). Despite progress in treatment and understanding of the disease, breast cancer is still the leading cause of cancer-related death in women (Bray et al. 2018). Risk factors include hereditary susceptibility due to mutations in particular genes, reproductive characteristics associated with estrogen and other hormones, pharmaceutical hormones, and lifestyle-related factors such as alcohol use and lack of exercise that affect hormone levels (Althuis et al. 2005). The incidence of hormone-dependent cancer has risen over the past 30 years in industrialized countries, which is unlikely to be only due to genetic predisposition. This has increased the research interest concerning gene–environment interactions that involve chemicals with hormone-like activity—often found in food, personal care products or as environmental contaminants—as potential factors for developing breast cancer (Colditz 1998).

These endocrine-disrupting chemicals (EDCs) have been shown to adversely affect the endocrine system in humans and other species. Some of the EDCs mimic endogenous estrogens by activating estrogen receptors (ERs), promoting mammary cell proliferation, and increasing the risk of initiating cell transformation and development of cancer (Sweeney et al. 2015; Diamanti-Kandarakis et al. 2009). Studies demonstrated that 79% of the EDCs that are able to promote carcinogenesis have estrogen-modulating effects related to carcinogenicity or mutagenicity (Choi et al. 2004; Yoon et al. 2014).

Perfluoroalkyl substances (PFAS) are a large group of environmental contaminants that have been produced since the 1950s and are used in many industrial and commercial applications, such as non-stick cookware, waterproof and breathable textiles, and food packing materials. PFAS are very resistant to biodegradation and are thus environmentally persistent (Dimitrov et al. 2004). Although perfluorooctane sulfonate (PFOS) and perfluorooctanoic acid (PFOA) now are banned in the European Union and the United States, they are still the most frequently detected PFAS, and ubiquitously found in human serum and breast milk (von Ehrenstein et al. 2009; Tao et al. 2008; Cariou et al. 2015). The exposure levels of PFAS vary among populations and over time. In blood, PFAS concentrations are often reported to be in the nanomolar range (Bartell et al. 2010; Karrman et al. 2007; Boronow et al. 2019). The tissue levels may be several times higher since the compounds are not metabolized in the body and are poorly eliminated (human half-life is estimated between 4 and 5 years) (Lau et al. 2007).

In vivo and in vitro studies have shown that PFAS have potential toxic effects. PFOS is suspected to be an EDC with estrogenic activity that may contribute to the risk of breast cancer (Jensen and Leffers 2008; Bonefeld-Jorgensen et al. 2011). Furthermore, in utero exposure to PFOA caused a significant increase in mammary fibroadenomas in the mouse dams and promoted mammary gland epithelial branching and growth in female pups (White et al. 2007). In addition, several studies involving the same species suggest that in utero and lactational exposure to PFOA delay development, and could increase the susceptibility of the mammary gland to carcinogens (White et al. 2009; Wolf et al. 2007; Macon et al. 2011; White et al. 2011). We have recently reported that both of these contaminants are able to induce human epithelial breast cell proliferation and neoplastic transformation via different mechanisms (Pierozan et al. 2018; Pierozan and Karlsson 2018). PFOS exposure promoted proliferation and migration/invasion in the human normal breast epithelial cells (MCF-10A) through alteration of regulatory cell-cycle protein levels and acceleration of the cell cycle via ER activation (Pierozan and Karlsson 2018). PFOA, on the other hand, induced cell-cycle dysregulation, cell proliferation and malignant cell transformation of MCF-10A cells through PPARα-dependent pathways (Pierozan et al. 2018). PFAS-induced epigenetic alterations may also be critically involved in these observed effects as epigenetic mechanisms such as DNA methylation and histone modifications are important processes implicated in tumorigenesis (Yamashita et al. 2018). Aberrant epigenetic regulation in breast cells can lead to the initiation, promotion, and maintenance of breast carcinogenesis, and is even implicated in playing an important role in the development of drug resistance (Lo and Sukumar 2008).

In the current study, we investigated the underlying mechanisms, including epigenetic modifications, of PFOS- and PFOA-induced breast epithelial cell transformation, and examined if the effects persist in the absence of the exposure, and are inherited from one cell generation to another for a multitude of cell divisions. This study can contribute to a better understanding of gene–environment interactions in the development of breast cancer.

## Materials and methods

### Chemicals

Dimethyl sulfoxide (DMSO), paraformaldehyde, 4´,6-diamidino-2-phenylindole dihydrochloride (DAPI), Triton X-100, propidium iodide (PI), PFOA, PFOS, cholera toxin, insulin, 3-(4,5-dimethyl2-yl)2,5-diphenyl-2H-tetrazolium bromide (MTT), epidermal growth factor (EGF) and hydrocortisone were obtained from Sigma-Aldrich (St Louis, MO, USA). Horse serum, penicillin–streptomycin (P/S), Dulbecco´s Phosphate-Buffered Saline (PBS), Dulbecco´s Modified Eagle´s Medium (DMEM) and trypsin solution (0.05%) were obtained from Gibco (Invitrogen, Paisley, UK). p53 monoclonal (DO-7), CDK6 monoclonal (75B9), CDK4 monoclonal (DCS-31), p21 monoclonal (R.229.6), phospho-Cyclin D1 (Thr 286) and occludin monoclonal (OC-3F10) antibodies as well as Syto 11 green fluorescent nuclei acid stain were obtained from Thermofisher Scientific (Rockford, IL, USA). p27 kip1 (D69C12), cyclin D1 (92G2), ERK 1/2 (137F5), phospho-ERK 1/2 (Thr202/Tyr204), JNK 1/2, phospho-JNK 1/2 (Thr183/Tyr185), p38, and phospho-p38 (Thr180/Tyr182) antibodies were obtained from Cell Signaling (Danvers, MA, USA). The secondary antibodies Alexa-Fluor 555 goat anti-mouse or 488 goat anti-rabbit IgG, and the blocking agent (normal goat serum) were obtained from Molecular Probes, Invitrogen (Paisley, UK). Matrigel Basement Membrane Matrix was obtained from Corning (New York, NY, USA). Histone H3 (EPR21228), histone H3 (acetyl K9), histone H3 (tri methyl K4), histone H3 (acetyl K27) (EP865Y), anti-E Cadherin (M168), anti-Integrin β1 (P5D2), anti-5-hydroxymethylcytosine (5-hmc), anti-5-methylcytosine (5-mc) and the secondary antibodies HRP-conjugated goat anti-rabbit and anti-mouse were obtained from Abcam (Cambridge, UK). Recombinant histone H3 (C110A) was obtained from Active Motif (Carlsbad, CA, USA).

### Cell culture

The human breast epithelial cells MCF-10A were obtained from the American Type Culture Collection (ATCC, Manassas, VA, USA). The cells were cultured as a monolayer in 10 cm^2^ tissue culture plastic flasks containing 10 ml of growth medium (DMEM/F12 supplemented with 5% horse serum, 20 ng/ml EGF, 0.5 mg/ml hydrocortisone, 100 ng/ml cholera toxin, 10 mg/ml insulin and 5 ml P/S). Cells were maintained at 37 °C and 5% CO_2_ in a humidified incubator.

### Exposure to PFOS and PFOA

**Fig. 1:**
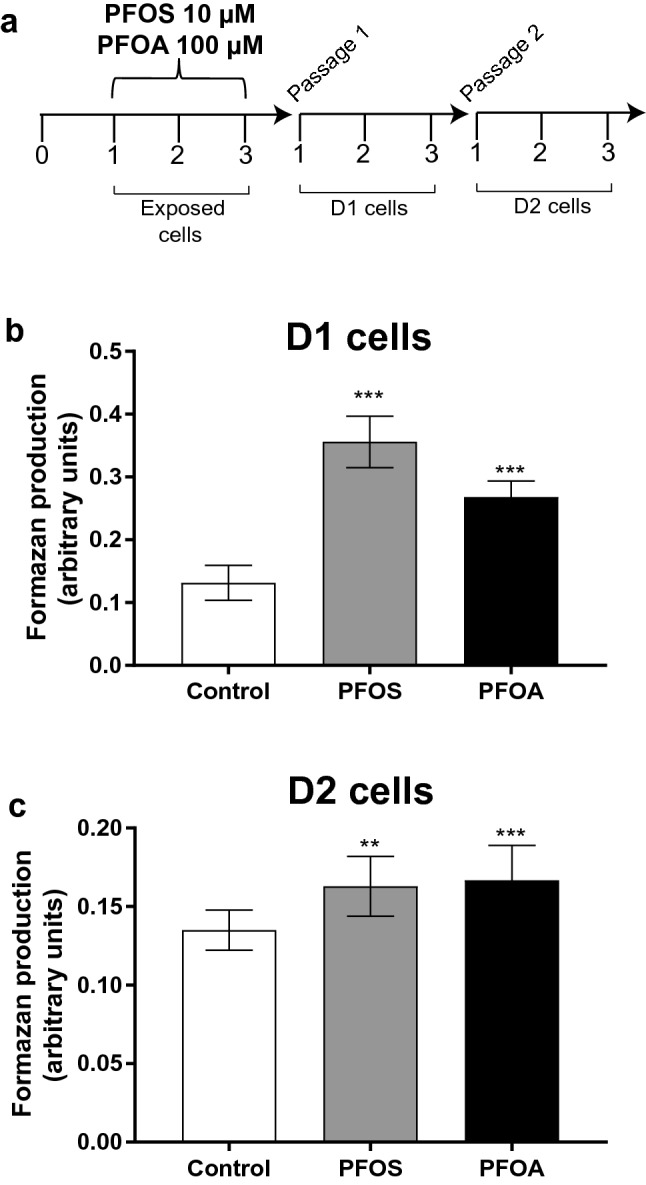
**a** Experimental model. After 1 day in culture, MCF-10A cells were exposed to PFOS (10 µM) or PFOA (100 µM) for 3 days and subcultured twice to study effects in unexposed daughter cells (D1 and D2). The MTT assay was used to measure cell proliferation according to Materials and methods. PFOS and PFOA increased the formazan production in both D1 (**b**) and D2 (**c**) cells. Values represent mean ± SD from three independent experiments. Statistically significant differences from control are indicated as follows: **p < 0.01 and ***p < 0.001 (One-Way ANOVA followed by the Tukey–Kramer test)

Cells were trypsinized and resuspended in the growth medium, and plated in 10 cm^2^ tissue culture plastic flasks (10^6^ cells/flask). One day after passaging, cells were treated with 10 µM PFOS or 100 µM PFOA for 72 h, dissolved in DMSO or water, respectively. Controls were exposed to 0.1% DMSO only. The concentrations were chosen based on previous studies where we showed that PFOS and PFOA were able to induce MCF-10A cell proliferation and cell invasion (Pierozan et al. 2018; Pierozan and Karlsson 2018). The exposed cells were then subcultured (one passage for daughter cells 1 (D1) and two passages for daughter cells 2 (D2)) by trypsin–EDTA incubation followed by washing, centrifugation, and plated at low density (500 cells/cm^2^). Mitotically heritable effects were then investigated in the unexposed D1 and D2 cells (Fig. 1a). The experiments were performed 3 days after each specific passages and repeated three times.

### Cell proliferation

MCF-10A cells were treated with 10 µM PFOS or 100 µM PFOA for 72 h and cell proliferation was measured in D1 and D2 cells using the MTT assay, as previously described (Pierozan et al. 2018). The absorbance was measured in 590 nm using a Spectramax i3 microplate reader (San Jose, CA, USA).

### Cell-cycle analysis

Cell-cycle analysis was conducted in the daughter cells by measuring the DNA content. Briefly, cells were fixed with 4% paraformaldehyde for 30 min and permeabilized with 0.1% Triton X-100. Cells were then incubated with DAPI (0.25 mg/ml) for 10 min and images were collected with a 10× objective in an ImageXpress Micro XLS Widefield High-Content Analysis System (Molecular Devices, Sunnyvale, CA, USA). Nine fields per well were automatically analyzed with the MetaXpress High-content image acquisition and analysis software after the digital acquisition, using the cell-cycle application module. Cells in different cell-cycle phases were presented as a percentage of the total number of cells counted.

### Immunocytochemistry

To evaluate the effects on cell-cycle regulatory proteins and adhesion proteins, immunocytochemistry was performed as previously described (Pierozan and Karlsson 2018). Briefly, cells were fixed with 4% paraformaldehyde for 30 min and permeabilized with 0.1% Triton X-100. Cells were then incubated overnight with primary antibodies (1:1000), followed by incubation with specific secondary antibodies conjugated with Alexa-fluor 488, 555 or 635 (1:500). Nuclei were stained with DAPI (0.25 mg/ml). Images were collected with a 10× objective in an ImageXpress Micro XLS Widefield High-Content Analysis System (Molecular Devices, Sunnyvale, CA, USA). Nine fields per well were automatically analyzed with the MetaXpress High-content image acquisition and analysis software after the digital acquisition, using the integrated fluorescence application module to analyze the intensity of the fluorescence. The nuclear levels of cyclin D1 were analyzed using the multi wavelength translocation module.

### Western blot

Cells were lysed with Laemmli lysis buffer and the protein concentration was determined by the Lowry assay (Lowry et al. 1951). An equal amount of protein was separated by sodium dodecyl sulfate–polyacrylamide gel electrophoresis (SDS-PAGE) on 4–20% gel and transferred to nitrocellulose membranes (Mini Trans-Blot Electrophoretic Transfer Cell; Bio-Rad, Hercules, CA, USA). The blot was then incubated in blocking solution (TBS; 500 mM NaCl, 20 mM Trizma, pH 7.8 with defatted dry milk), followed by washes with TBS and incubation overnight in TBS containing monoclonal antibodies (phospho-Cyclin D1, total Cyclin D1, phospho-ERK, total ERK, phospho-p38, total p38, phospho-JNK, total JNK) diluted 1:5000. The blots were then washed with TBS and incubated for 1 h in TBS containing peroxidase-conjugated mouse anti-rabbit IgG diluted 1:10,000. The blot was developed with the chemiluminescence ECL kit (Bio-rad, Hercules, CA, USA) using a charge-coupled device (CCD) imager (Thermofisher, Rockford, IL, USA), and optical density was measured using the ImageJ Software. The results were normalized by the β-tubulin content and the phospho/total ratio of the proteins was expressed as a percentage of the control.

### Malignance analysis

#### Migration and invasion assay

Transwell migration and invasion assays were conducted as previously described (Pierozan et al. 2018). The daughter cells of MCF-10A cells exposed to PFOS and PFOA were plated in the upper chamber of transwells without (migration assay) or with 200 µg/ml Matrigel Matrix (invasion assay), and the lower chamber contained 100 µl growth medium. Cells were incubated for 24 h at 37 °C in a humidified incubator. After that, the upper chamber was removed and invasive cells in the bottom were stained with DAPI, imaged with ImageXpress Micro XLS Widefield High-Content Analysis System, and analyzed with the MetaXpress High-content image acquisition and analysis software (Molecular Devices, Sunnyvale, CA, USA), using the cell counting module.

#### Markers of malignancy

To evaluate some malignancy markers, immunocytochemistry was performed as described above in the invasive cells that were able to migrate during the invasion assay. Cells were marked with an anti-E Cadherin antibody, anti-Integrin β1 antibody and anti-occludin antibody, and incubated with specific secondary antibodies conjugated to Alexa-fluor 488, 635 or 555. Images were collected with a 10x objective in an ImageXpress Micro XLS Widefield High-Content Analysis System (Molecular Devices, Sunnyvale, CA, USA). Nine fields per well were automatically analyzed with the MetaXpress High-content image acquisition and analysis software (Molecular Devices, Sunnyvale, CA, USA), using the integrated fluorescence application mode.

### Histone modification analysis

Total histones were extracted from the samples using the high-salt extraction protocol (Shechter et al. 2007) with some modifications. Briefly, cells were re-suspended in extraction buffer (10 mM HEPES, 10 mM KCl, 1.5 mM MgCl_2_, 0.34 M sucrose, 10% glycerol and 0.2% NP-40), centrifuged at 6500 g for 10 min and the isolated nuclei were lysed in non-salt buffer (3 mM EDTA, 0.2 mM EGTA). The samples were centrifugated at 6500 for 10 min and the chromatin pellet was re-suspended in a high-salt buffer (50 mM Tris–HCl, 2.5 M NaCl and 0.05% NP-40). The protein content was measured by the Bradford assay (Bradford 1976) and the samples were prepared for western blot assay as described above. A recombinant histone H3 protein was used as positive control and the levels of the specific histone modifications (H3K9ac, H3K9me2, H3K27ac, and H3K4me3) were normalized by the total H3 content and expressed as a percentage of the control.

### Global DNA methylation

DNA was extracted from cells using the AllPrep DNA/RNA micro kit (Qiagen, Germany). The concentration of DNA was measured using a Nanodrop 2000 spectrophotometer (Thermo Scientific, USA). The concentration of 5-methyl cytosine was quantified by ELISA. The negative control used was unmethylated DNA (Active motif, Carlsbad, CA, USA) and the standard curve was prepared using methylated DNA (Active motif, Carlsbad, CA, USA). PBS and 100 ng of each DNA sample was added to PCR tubes to a final volume of 100 µl. The DNA samples were denatured by heating at 98 °C for 5 min and then transferred immediately to ice for 10 min. Then, the DNA samples were added to 96-well microtiter plate, covered with aluminum foil and incubated at 37 °C for 1 h. After the incubation, the liquid of the wells was discarded and the wells were washed 3 times with 200 µl washing buffer (PBS/0.2% Tween-20). The plates were then blocked with a blocking buffer (Thermo Pierce, Rockford, IL, USA) and incubated at 37 °C for 30 min. An antibody mixture was prepared with anti-5-methylcytosine in PBS and incubated overnight at 4 °C. After the incubation, the wells were washed three times with washing buffer, followed by incubation with secondary antibody HRP-conjugated for 30 min. The wells were then washed three times with washing buffer, and the 3,3′,5,5′- tetramethylbenzidine (TMB) substrate solution (Thermo Pierce, Rockford, IL, USA) was added to the plate in a volume of 50 µl per well. The color reaction was stopped by the addition of 50 µl of stop solution (Thermo Pierce, Rockford, IL, USA) and optical density was measured at 450 nm using a Spectramax i3 microplate reader (San Jose, CA, USA).

### Statistical analysis

The results are presented as mean ± standard deviation (SD). Data from the experiments were analyzed by One-Way ANOVA followed by the Tukey–Kramer test when the F test was significant using the GraphPad Prism 7 software. P < 0.05 was considered significant.

## Results

### Persistent effects of PFOS and PFOA on MCF-10A cell proliferation

Mitotically inherited effects of PFOS and PFOA exposure were investigated by exposing MCF-10A cells to concentrations previously shown to increase proliferation in the exposed cells (Pierozan et al. 2018; Pierozan and Karlsson 2018) and analyzing unexposed daughter cells after one and two passages (D1 and D2 cells). Each passage includes approximately 7000 cell divisions (Fig. 1a). The results demonstrated an increase in formazan production in both D1 and D2 cells compared to the respective control group (Fig. 1b, c).

The analysis of cell-cycle distribution revealed an accumulation of D1 and D2 cells in S (both compounds) and G2/M phases (PFOA in D1 and PFOS in D2) (Table 1), suggesting that the compounds alter cell programming in MCF-10A cells and continue to promote cell proliferation through cell-cycle progression after a multitude of cell divisions.

**Table 1:** Effects on the cell cycle in daughter cells (D1 and D2) of MCF-10A cells exposed to PFOS (10 µM) or PFOA (100 µM)

D1	G0/1	S	G2/M
CONTROL	69.96 ± 3.08	16.64 ± 0.98	7.58 ± 2.89
PFOS	35.54 ± 7.63***	38.19 ± 0.84***	14.11 ± 7.53
PFOA	36 ± 4.66***	37.76 ± 0.56***	22.97 ± 4.64***
D2	G0/1	S	G2/M
CONTROL	77.71 ± 4.75	16.51 ± 1.2	5.78 ± 4.39
PFOS	56.36 ± 10.65***	23.15 ± 0.3***	20.49 ± 10.8**
PFOA	65.8 ± 4.43*	23.83 ± 1.66***	10.37 ± 4.13

Results are shown as percentage of total events (10,000 cells). Statistically significant differences from control are indicated as follow: ***P < 0.001; **P < 0.01 and *P < 0.05 (One-Way ANOVA followed by the Tukey–Kramer test)

### PFOS and PFOA alter the levels of regulatory cell-cycle proteins in the unexposed daughter cells

To investigate the mechanisms involved in PFOS and PFOA-induced cell proliferation and alteration of the cell cycle in the daughter cells, the levels of cyclin-dependent kinases (CDK4, CDK6, Cyclin D1) and their respective inhibitors (p27, p21 and p53), as well as some enzymes involved in cell-cycle regulation (ERK, JNK and p38) were analyzed.

**Fig. 2:**
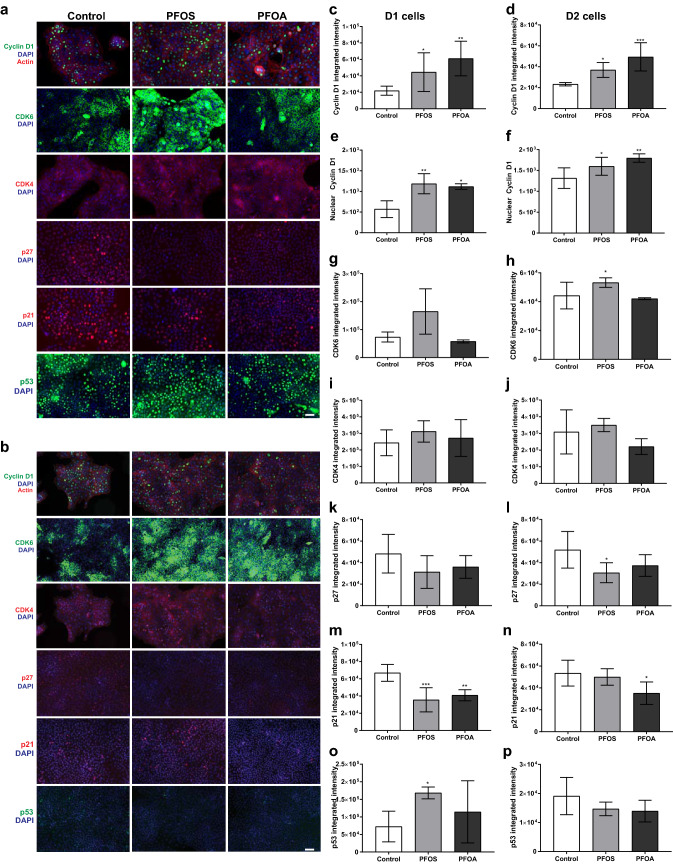
Effects on regulatory cell-cycle proteins in daughter cells (D1 and D2) of MCF-10A cells exposed to PFOS (10 µM) or PFOA (100 µM). Representative images of D1 (**a**) and D2 (**b**) cells immunostained with Cyclin D1 and actin, CDK6, CDK4, p27, p21 and p53. Integrated fluorescence intensity (**c**–**d** and **g**–**p**) and nuclear cyclin D1 levels (**e**, **f**) were analyzed as described in Materials and methods. Values represent mean ± SD from three independent experiments. Statistically significant differences from control are indicated as follows: *p < 0.05, **p < 0.01 and ***p < 0.001 (One-Way ANOVA followed by the Tukey–Kramer test). Scale bar = 50 µm

Representative fluorescence microscopy images are shown in Fig. 2a (D1), b (D2). The image analysis revealed that the treatment of the MCF-10A cells with PFOS or PFOA caused an increase in total cyclin D1 levels (Fig. 2c, d), as well as nuclear levels in both D1 and D2 cells (Fig. 2e, f). The levels of CDK6 were not altered by PFOS or PFOA in D1 cells (Fig. 2g), while D2 cells derived from PFOS exposed cells demonstrated an increase in the levels of this enzyme (Fig. 2h). No alteration was observed in the CDK4 levels in D1 (Fig. 2i) or D2 cells (Fig. 2j) for any of the compounds. The p27 levels were decreased by PFOS in D2 cells (Fig. 2k, l), while both compounds decreased the p21 levels in D1 cells (Fig. 2m); this effect only persisted in PFOA D2 cells (Fig. 2n). The levels of p53 were specifically increased in PFOS D1 cells (Fig. 2o, p).

**Fig. 3:**
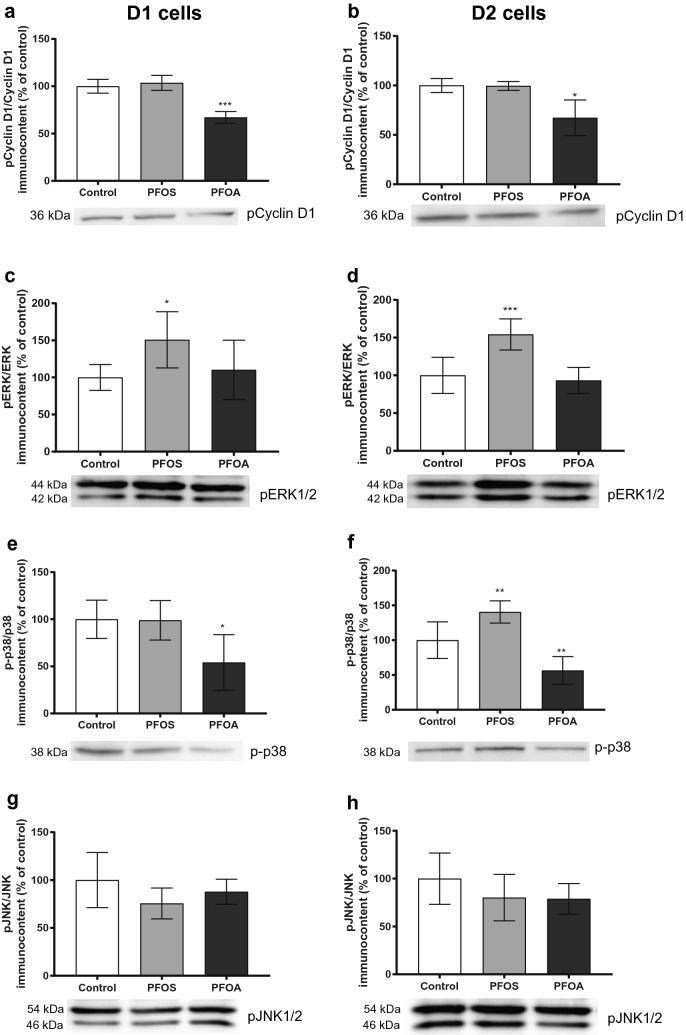
Involvement of phosphorylated cyclin D1 and MAPK in the effects triggered by PFOS (10 µM) and PFOA (100 µM) in the daughter cells (D1 and D2). Phospho-cyclin D1/cyclin D1 (**a**, **b**), phospho-ERK/ERK (**c**, **d**), phospho-p38/p38 (**e**, **f**) and phospho-JNK/JNK (**g**, **h**) protein levels in MCF-10A cells. β-tubulin was used as a loading control. Representative blots of three experiments are shown. Values represent mean ± SD from three independent experiments. Statistically significant differences from control are indicated as follows: *p < 0.05, **p < 0.01 and ***p < 0.001 (One-Way ANOVA followed by the Tukey–Kramer test)

To further investigate the mechanisms by which the compounds alter the cell-cycle regulatory proteins, the phosphorylated levels of cyclin D1 (thr286), ERK1/2 (Thr202/Tyr204), p38 (Thr180/Tyr182) and JNK1/2 (Thr183/Tyr185) were analyzed by western blot (Fig. 3). The results showed that, for PFOA, the levels of phosphorylated cyclin D1 at thr286 were decreased in both, D1 and D2 cells (Fig. 3a, b). No alteration of phosphorylated cyclin D1 was observed in the daughter cells derived from the MCF-10A cells treated with PFOS. This compound was instead found to increase the phosphorylation of ERK1/2 in both D1 and D2 cells (Fig. 3c, d). The levels of phosphorylated p38 were decreased in PFOA D1 and D2 cells, while PFOS D2 cells demonstrated an increase of phosphorylated p38 (Fig. 3e, f). No alterations were observed in the levels of phosphorylated JNK for any of the compounds or cell passages (Fig. 3g, h).

### PFOS and PFOA caused a persistent malignant transformation in the unexposed daughter cells

**Fig. 4:**
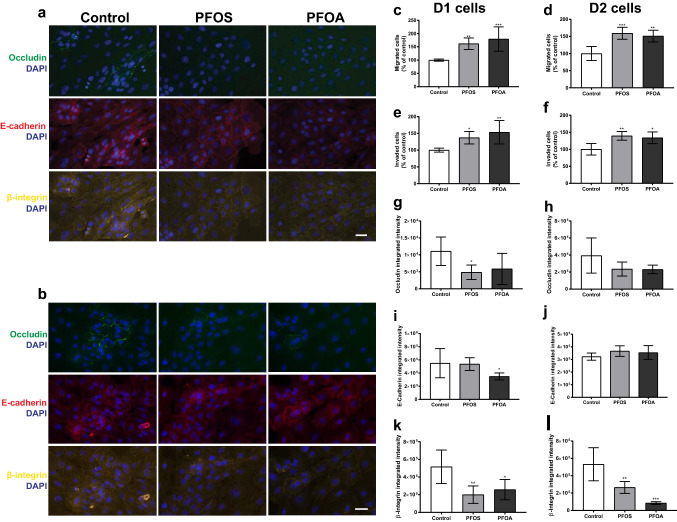
Persistent effects on cell migration and cell adhesion proteins in daughter cells (D1 and D2) of MCF-10A cells exposed to PFOS (10 µM) or PFOA (100 µM). Representative images of D1 (**a**) and D2 (**b**) cells immunostained with DAPI, occludin, E-cadherin and β-integrin. Transwell migration (**c**, **d**), matrigel invasion (**e**, **f**) and integrated fluorescence intensity of invaded cells (**g**–**l**) were measured as described in Materials and methods section. Values represent mean ± SD from three independent experiments. Statistically significant differences from control are indicated as follows: *p < 0.05, **p < 0.01 and ***p < 0.001 (One-Way ANOVA followed by the Tukey–Kramer test). Scale bar = 30 µm

The persistent effects of PFOS and PFOA on cell proliferation prompted us to investigate if the increase in cell migration and invasion also persists in the unexposed daughter cells after a multitude of cell divisions (Fig. 4). Representative fluorescent images are shown in Fig. 4a (D1 cells) and b (D2 cells). The results showed that both compounds caused a persistent cell transformation that promotes cell migration (Fig. 4c, d) and invasion (Fig. 4e, f) in both D1 and D2 cells.

Loss of cell–cell and cell–extracellular matrix adhesion molecules are prerequisites for malignant tumor cells to dissociate from the primary tumor mass and invade the surrounding stroma (Behrens 1993). To investigate the mechanisms by which the compounds induced MCF-10A cell transformation, we evaluated the levels of some adhesion proteins in the invading cells. The occludin levels were found to be decreased in PFOS D1 cells, but the effect did not persist in D2 cells. No alterations of occludin levels were observed in the PFOA daughter cells (Fig. 4g, h). The levels of E-cadherin were only altered in D1 cells derived from PFOA-exposed cells (Fig. 4i. j). Both contaminants induced a persistent decrease in the β-integrin levels in D1 and D2 cells (Fig. 4k, l).

### PFOS- and PFOA-induced epigenetic alterations

**Fig. 5:**
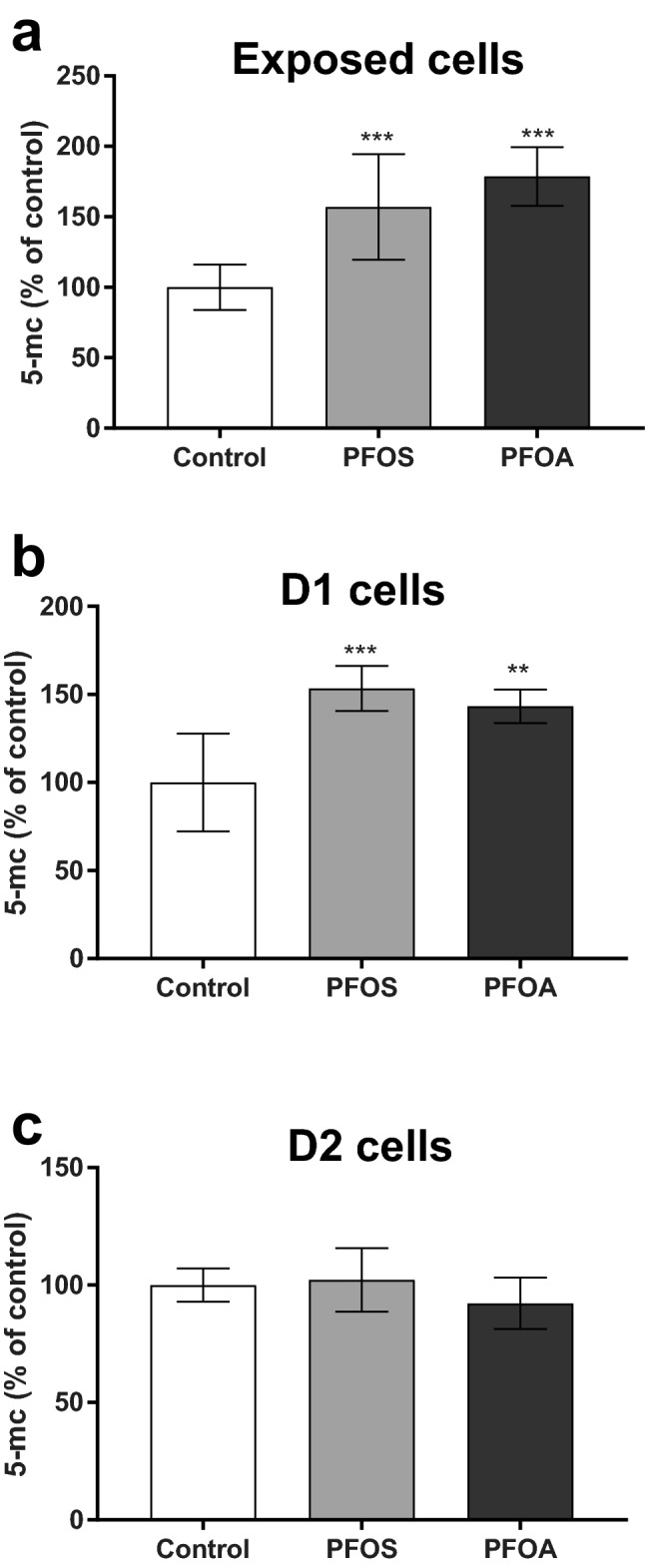
PFOS (10 µM) and PFOA (100 µM) increased global DNA methylation in the exposed cells and D1 daughter cells. Total DNA methylation in MCF-10A cells was determined by measuring 5-methylcytosine (5-mc) in the exposed (**a**), D1 (**b**) and D2 (**c**) cells as described in the Materials and methods. Values represent mean ± SD from three independent experiments. Statistically significant differences from control are indicated as follows: **p < 0.01 and ***p < 0.001 (One-Way ANOVA followed by the Tukey–Kramer test)

To investigate if the PFOS- and PFOA-induced effects on proliferation and cell transformation in the D1 and D2 cells may involve epigenetic mechanisms, global histone modification and DNA methylation were analyzed in the exposed cells and daughter cells. Both compounds were found to increase the global DNA methylation in exposed cells (Fig. 5a) and D1 cells (Fig. 5b). However, the effect did not persist in D2 cells (Fig. 5c).

**Fig. 6:**
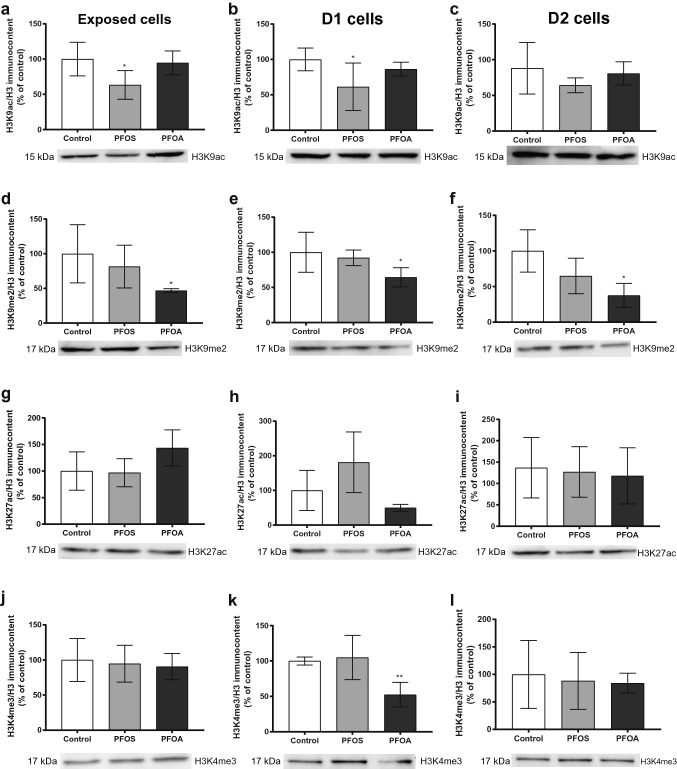
PFOS (10 µM) and PFOA (100 µM) altered global histone modification in the exposed cells and daughter cells (D1 and D2). Protein levels of H3K9ac (**a**–**c**), H3K9me2 (**d**–**f**), H3K27ac (**g**–**i**) and H3K4me3 (**j**–**l**) were determined by western blotting as described in Materials and methods. Representative blots are shown. Values represent mean ± SD from three independent experiments. Statistically significant differences from control are indicated as follows: *p < 0.05; **p < 0.01 (One-Way ANOVA followed by the Tukey–Kramer test)

The levels of acetylated H3K9 (H3K9ac) were decreased in PFOS-treated cells and D1 cells (Fig. 6a, b), but the effect did not persist in the D2 cells (Fig. 6c). The levels of dimethylated H3K9 (H3K9me2) were decreased in PFOA-treated cells, and this effect were also observed in both D1 and D2 cells. PFOS did not induce any similar alteration of H3K9me2 levels (Fig. 6d–f). The levels of acetylated H3K27 (H3K27ac) were not modified by the treatment with PFOS or PFOA (Fig. 6g–i). A decrease in trimethylated H3K4 (H3K4me3) levels was observed only in PFOA D1 cells (Fig. 6j–l).

## Discussion

Breast cancer was one of the three most common cancer types in terms of incidence and ranked as fifth in terms of mortality worldwide, in 2018 (Bray et al. 2018). Here, we demonstrated that the ubiquitous contaminants PFOS and PFOA are able to cause increased proliferation of human breast epithelial cells, which persisted after a multitude of cell divisions, by differentially affecting regulatory cell-cycle proteins. In addition, both contaminants induced an increased global DNA methylation, altered histone modifications, and decreased levels of cell adhesion proteins involved in cell–cell and cell–extracellular matrix adhesion, leading to cell migration and invasion. Loss of control at the G1-to-S transition is a hallmark of tumor development, and aberrant cyclin D1 expression is reported in many human cancers (Vermeulen et al. 2003). Cyclin D1 is a sensor of extracellular signals and plays a key role in G1–S phase progression (Alt et al. 2000). Both PFOS and PFOA caused a persistent increase in cyclin D1 levels, which could explain the observed increase in cell proliferation. Interestingly, the compounds increased the levels of this key protein through two different mechanisms. Exposure of MCF-10A cells to PFOS caused activation of ERK in the unexposed daughter cells. The ERK pathway is activated by mitogen factors, and is one of the most mutated genes in various cancers, often leading to an increase in cell proliferation (Samatar and Poulikakos 2014). Activated ERK translocates from the cytoplasm to the nucleus, where it phosphorylates and activates several nuclear targets, including transcription factors such as AP-1 that binds to the cyclin D1 promoter and increase the transcription of cyclin D1 (Karin 1996). PFOA, on the other hand, increased the levels of cyclin D1 not by increasing its transcription, but by inactivating p38 and decreasing cyclin D1 degradation. p38 negatively regulates cell-cycle progression both at the G1/S and the G2/M transition by several mechanisms, including downregulation of cyclins and modulation of p53 (Ambrosino and Nebreda 2001; Thornton and Rincon 2009). Phosphorylation of cyclin D1 at Thr286 by p38 promotes the nuclear-to-cytoplasmic redistribution of cyclin D1 during S phase of the cell cycle, and its subsequent degradation in the cytoplasm (Diehl et al. 1998). In addition to the above-mentioned mechanisms, both PFOS and PFOA decreased the cellular levels of the CDK inhibitor p21. Decreased expression of cell-cycle inhibitors is associated with promotion of tumor formation and poor prognosis in many types of cancers (Burton et al. 2000). p21 suppresses tumorigenesis by promoting cell-cycle arrest in response to various stimuli, and substantial evidence indicates that p21 acts as a master effector of multiple tumor suppressor pathways (Abbas and Dutta 2009). PFOS also decreased the levels of the cell-cycle inhibitor p27, but only in the D2 cells. This tumor suppressor gene is frequently inactivated in human breast cancer, and loss of p27 is an indicator of poor patient outcome in a majority of breast cancer studies (Alkarain et al. 2004). Inactivation of p27 in human cancer is suggested to rarely be due to mutation, but instead mainly occurs at post-translational levels, via protein degradation, mislocalization and/or sequestration (Belletti and Baldassarre 2012).

In many cancer-related deaths, it is not the primary tumor but its metastases that are the main cause of death (Weigelt et al. 2005). Breast cancer cells can spread to distant tissues through metastases a long time after the primary tumor developed. The first step towards metastasis is an invasion or directed migration of tumor cells into adjacent tissues. To be able to invade neighboring tissues and metastasize, the invasive tumor cells must first alter their cell–cell adhesion and cell adhesion to the extracellular matrix (Scully et al. 2012). Our results show that PFOS and PFOA alter the MCF-10A cells phenotype from normal to malignant, at least in part, by decreasing the occludin, E-cadherin and β-integrin levels, consequently altering the cell–cell and cell–extracellular membrane interactions. Adhesion molecules, such as integrins and the cadherin complex, seem to be the key components of tumor invasion (Gerashchenko et al. 2019). Down-regulation of E-cadherin is shown to be important for breast cancer metastasis and may reflect the progression and metastasis in breast cancer associated with poor prognosis (Lambert et al. 2017; Onder et al. 2008). The integrin family regulates a diverse array of cellular functions crucial to the initiation, progression, and metastasis of tumors, and decreased expression of integrin contributes to the altered adhesive properties of tumors cells characteristic of a malignant phenotype which has been reported in several breast cancer types (Desgrosellier and Cheresh 2010; Zutter et al. 1990; Zutter et al. 1993). Studies have also demonstrated that the integral membrane protein occludin is significantly decreased in metastatic breast cancer (Martin et al. 2010) and its overexpression enhanced cellular sensitivity to apoptotic stimuli and suppressed tumor development (Osanai et al. 2006).

Growing evidence shows that epigenetic mechanisms such as DNA methylation, histone modifications and nucleosome remodeling play a key role in carcinogenesis (Yamashita et al. 2018; Jovanovic et al. 2010). Altered expression of key genes in breast cells through aberrant epigenetic regulation can lead to the initiation, promotion, and maintenance of carcinogenesis (Lo and Sukumar 2008). Both PFOS and PFOA induced an increased global DNA methylation in the exposed MCF-10A cells and unexposed daughter cells. In cancer cells, CpG islands that are normally unmethylated can become methylated, which may result in the silencing of important genes (Jovanovic et al. 2010), such as tumor-suppressor genes, genes that suppress tumor invasion and metastasis, DNA repair genes and genes that inhibit angiogenesis (Lo and Sukumar 2008; Esteller et al. 2000; Radpour et al. 2011). However, more studies are necessary to identify the specific genes that are hypermethylated and potentially silenced by these two contaminants. CpG-island hypermethylation in cancer cells is associated with the deacetylation of histones H3 and H4 (Kondo 2009). Altered histone modifications can affect the structure and integrity of the genome and disrupt normal patterns of gene expression (Jovanovic et al. 2010). Acetylation at lysine 9 (H3K9ac) and 27 (H3K27ac) of histone H3 and methylation at K4 (H3K4me) are modifications generally associated with open chromatin structure and active gene transcription (Esteller 2007). While, mono, di, and tri-methylation of H3K9 are implicated in closed chromatin structure and gene silencing (Baylin and Ohm 2006). PFOA was found to decrease H3K9me2 levels in the exposed cells and this effect persisted in the D1 and D2 cells. The reduction of H3K9me2 levels has been demonstrated to alter the expression of genes involved in breast cancer transformation (Zhao et al. 2016). PFOS, on the other hand, caused a decrease in H3K9 acetylation levels which have been shown to be reduced in breast cancer, as well as other cancers, and is correlated with both tumor progression and poor clinical outcomes (Elsheikh et al. 2009). The PFAS-induced epigenetic alterations may be directly linked to the observed effects on cell-cycle proteins. For example, methylation of the promoter region and alteration to an inactive chromatin by histone deacetylation are important mechanisms involved in the inactivation of p21 (Fang and Lu 2002). Studies also show that histone deacetylation inhibitors induced melanoma cell growth arrest by upregulating p21 and p27 (Cheng et al. 2019).

**Fig. 7:**
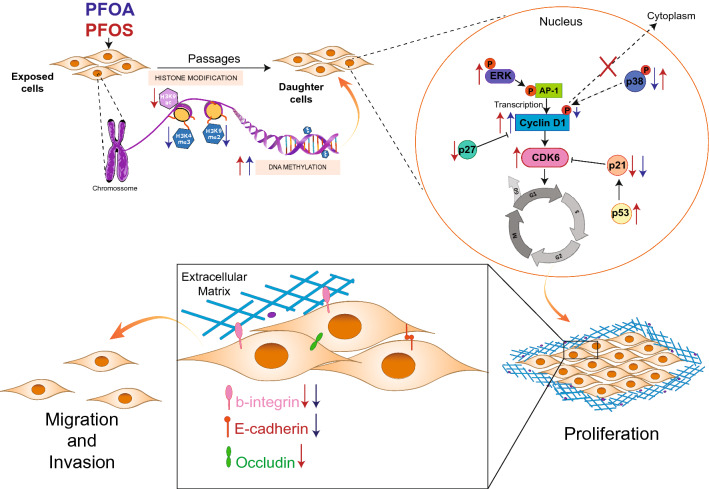
Schematic model of how PFOS and PFOA may promote persistent cell-cycle progression and stimulate transformation of MCF-10A cells. PFOS and PFOA up-regulate cyclin D1 through different mechanisms, and down-regulate p21 to drive cells from G1 to S, promoting cell-cycle progression and cell proliferation. The compounds also decreased the levels of occludin, β-integrin and E-cadherin, which promote the migration and invasion capacity. The underlying molecular pathways may involve epigenetic mechanisms as both compounds induced an increased global DNA methylation and altered important histone modifications

In summary, this study demonstrates that exposure of human breast epithelial cells to PFOA and PFOS induced an increase in cell proliferation, cell migration and invasion potential, by differentially affecting proteins such, cell-cycle regulators, β-integrin, E-cadherin and occludin as well as global DNA methylation and histone modifications (Fig. 7). It is worth pointing out that despite the persistent effects on cell proliferation and migration/invasion capacity, the alterations of the regulatory enzymes and epigenetic modifications vary between D1 and D2 cells, particularly for PFOS, indicating that the molecular pathways underlying the malignant phenotype change after numerous cell divisions. Increased knowledge of these contaminant-induced effects and their contribution to breast tumorigenesis is important for a better understanding of gene–environment interactions in the etiology of breast cancer.
